# Dual-regulatory miRNAs: master regulators and therapeutic targets in bone-metastatic breast cancer

**DOI:** 10.3389/fmolb.2025.1680908

**Published:** 2025-10-28

**Authors:** Feifei Meng, Mengdi Zhang, Dongqing Pu, Guangxi Shi, Jingwei Li

**Affiliations:** ^1^ College of First Clinical Medicine, Shandong University of Traditional Chinese Medicine, Jinan, China; ^2^ Department of Breast and Thyroid Surgery, Affiliated Hospital of Shandong University of Traditional Chinese Medicine, Jinan, China

**Keywords:** miRNAs, breast cancer, bone metastasis, targeted therapy, bone microenvironment

## Abstract

Breast cancer bone metastasis involves dynamic reprogramming of transcriptional networks and cellular homeostasis. Current primary treatment strategy relies on palliative care, and the search for effective therapeutic targets remains a critical challenge. MicroRNAs (miRNAs), endogenous non-coding RNA molecules, exert precise regulation of gene expression through sequence-specific binding to the 3′ UTR of target mRNAs. Accumulating evidence has established miRNAs as pivotal regulators of breast cancer and its metastatic bone disease. Depending on their target genes, individual miRNAs may function as oncogenic miRNAs (oncomiRs) or as tumor suppressor miRNAs (tsmiRs), and hold potential as biomarkers for diagnosis and prognosis. This review systematically analyzes the regulatory mechanisms of critical miRNAs and their target genes in breast cancer bone metastasis, offering novel insights for early diagnosis and targeted therapeutic strategies.

## Graphical Abstract

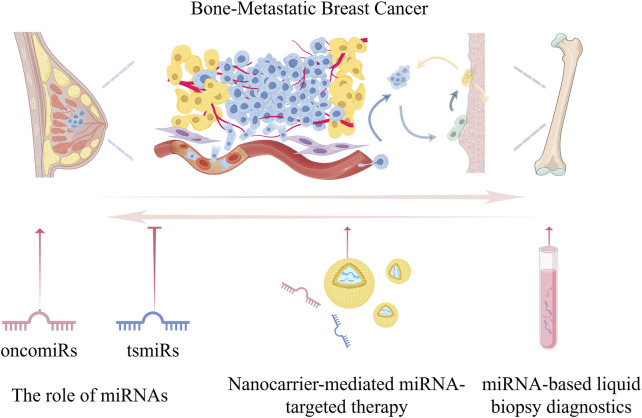

## 1 Introduction

Breast cancer remains the most prevalent malignancy among women worldwide, with bone metastasis representing the most common site of distant metastasis (Sui et al., 2022). Epidemiological data reveal that approximately 90% of breast cancer-related deaths are caused by complications arising from metastatic disease. Notably, bone metastasis occurs in up to 70% of metastatic breast cancer patients (Chen et al., 2018). These skeletal lesions frequently precipitate severe skeletal-related events, including intense bone pain, spinal cord or nerve compression, hypercalcemia, urinary and bowel dysfunction, and even paralysis, all of which contribute to elevated mortality rates (Aielli and Ponzetti Mrucci, 2019). Current therapeutic strategies include systemic therapies, such as chemotherapy and endocrine treatment, aimed at inhibiting tumor proliferation, as well as bone-targeted drugs, such as bisphosphonates or denosumab, which inhibit excessive cancer-induced bone destruction (Coleman et al., 2014). However, while these interventions alleviate symptoms and improve quality of life, they remain palliative, underscoring the need for new therapeutic or diagnostic interventions to prevent the formation of bone metastasis.

In recent years, the underlying mechanisms of bone metastasis have become a popular research focus, with increasing evidence supporting the “seed and soil” hypothesis, which posits that tumor cells can only thrive in a microenvironment that is conducive to their growth (Marazzi et al., 2020; Mashouri et al., 2019; Elshimy et al., 2025). Breast cancer bone metastasis-a complex, multi-step process (Liang et al., 2020; Shin Ekoo and Koo, 2020; Arakil et al., 2024)-often begins with clinically undetectable micrometastases preceding overt lesions, involving the formation and invasion of the primary breast tumor, the survival of tumor cells along the metastatic pathway, and their colonization and proliferation at the bone site, making vigilant monitoring and early intervention crucial. Currently, effective and reliable markers for monitoring and therapeutic targets for detecting and (Arakil et al., 2024) treating breast cancer bone metastasis remain lacking in clinical trials (Arakil et al., 2024), highlighting the urgent need for foundational research to elucidate the underlying mechanisms and molecular networks.

Significantly, microRNAs (miRNAs) have emerged as crucial post-transcriptional regulators in this process, capable of accurately controlling the expression of various genes linked to tumor invasion, bone colonization, and osteoclast activation. By orchestrating these intricate interactions, miRNAs occupy a central role within the signaling network of metastasis. miRNAs are small endogenous non-coding RNAs comprising 19–25 nucleotides. In 1993, Victor Ambros and colleagues discovered that the *lin-4* gene, the first identified miRNA, regulates developmental timing in *Caenorhabditis elegans* (Slack and Ruvkun, 1997; Lee et al., 1993). Subsequent studies have shown that miRNAs are widely expressed across human tissues and organs, playing critical roles in physiological processes (Abadi et al., 2021) such as proliferation, apoptosis, differentiation, and metabolism, as well as in pathological processes like cancer, pulmonary fibrosis, and diabetes. In recent years, the aberrant expression of miRNAs has been increasingly associated with the progression of human breast cancer. This review summarizes recent findings on key miRNAs involved in the formation and development of breast cancer bone metastasis, aiming to provide a theoretical foundation for understanding its molecular regulatory network and identifying new therapeutic targets for clinical diagnosis and treatment.

## 2 Biogenesis and function of miRNAs

**FIGURE 1:**
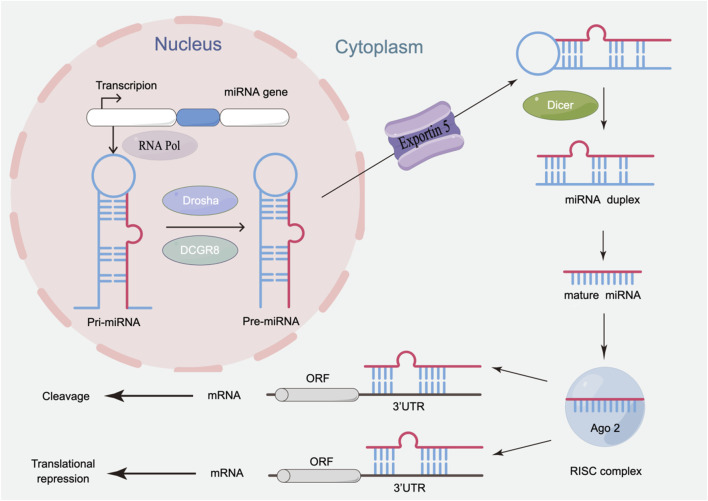
Biosynthesis process of miRNA.

MiRNA biogenesis involves tightly regulated nuclear and cytoplasmic processing stages (Figure 1). Initially, miRNA genes are transcribed into primary miRNAs (pri-miRNAs) by RNA polymerase II (RNA Pol II) in the nucleus. The pri-miRNAs are then processed by the nuclease Drosha and its cofactor DGCR8 to form precursor miRNAs (pre-miRNAs) (Hill and Tran, 2021). Exportin-5 mediates the translocation of pre-miRNA to the cytoplasm, where the Dicer enzyme catalyzes the excision of the terminal loop to generate mature double-stranded miRNAs (Bernstein et al., 2001). These mature miRNAs are loaded onto Argonaute (AGO) proteins, forming the miRNA-induced silencing complex (miRISC). One strand is rapidly degraded, while the other strand can bind to the 3′ untranslated region (UTR) of target mRNAs, inhibiting translation or inducing specific degradation of mRNA, thereby influencing post-transcriptional regulation of target genes, and participating in key cellular processes.

The function of miRNAs primarily depends on their ability to bind to the 3′ UTR of target mRNAs, thereby regulating gene expression (Desvignes et al., 2021). Interestingly, a single miRNA can target multiple different mRNA targets, and the expression of specific mRNAs can be regulated by several distinct miRNAs. Thus, miRNA-mediated gene regulation affects nearly every fundamental cellular process (Jia and Wei, 2020). Furthermore, cancer-related miRNAs are broadly categorized as oncogenic miRNAs (oncomiRs), which promote tumorigenesis by suppressing tumor suppressor genes, or tumor suppressor miRNAs (tsmiRs), which inhibit oncogene expression to counteract malignant progression (Loh et al., 2019). Studies have also shown that in bone metastasis, abnormal miRNA expression, as a crucial driver of intracellular molecular changes, participates in regulating the pathological processes of bone metastasis in various cancers, including breast cancer (Puppo et al., 2021). Therefore, miRNAs could serve as promising therapeutic targets and regulators of metastatic progression in breast cancer bone metastasis.

## 3 miRNAs in the bone microenvironment of breast cancer metastasis

**FIGURE 2:**
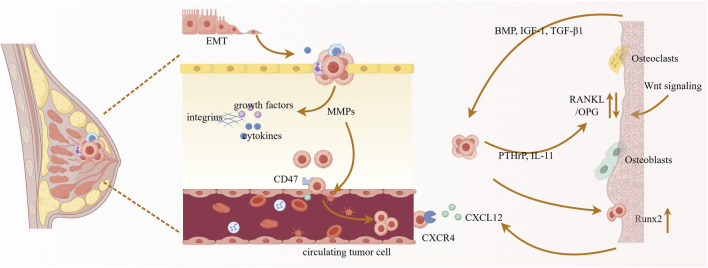
Bone metastasis process of breast cancer.

Bone metastasis is a pathological connection between the primary tumor and the secondary metastatic site in bone, arising from reciprocal interactions between disseminated tumor cells and the bone microenvironment. Tumor metastasis is a multi-step cascade process (Figure 2) involving tumor cell invasion and infiltration, intravasation across the vascular endothelium into the circulatory system, survival in circulation, subsequent extravasation to distant organs, adaptation to the metastatic microenvironment, and proliferation to form metastatic lesions (Liang et al., 2020). Epithelial-mesenchymal transition (EMT), as a key step, confers invasiveness to tumor cells, facilitating their dissemination to distant sites (Hanahan and Folkman, 1996; Huber and Kraut Nbeug, 2005). Secondly, the matrix metalloproteinase (MMP) family of extracellular proteases, secreted by tumor cells, degrades the basement membrane and extracellular matrix, which also leads to the release of growth factors and cytokines from cells and the extracellular matrix, thereby promoting cancer cell growth and survival (Liang et al., 2020; Kessenbrock and Plaks Vwerb, 2010). Subsequently, abnormally structured blood vessels within the tumor tissue (such as leaky vasculature) (Weis and Mcheresh, 2011) facilitate tumor cell entry into the bloodstream; circulating tumor cells can express CD47 (Chao et al., 2012), among other molecules, to evade phagocytosis by macrophages. Metastatic breast cancer cells migrate via the bloodstream from the primary site to distant organs such as the bone (Song and Wei Cli, 2022). Bone cells can release factors like CXCL12 to promote cancer cell bone metastasis, and the abundant blood supply and continuous remodeling process in bone create a permissive microenvironment: osteoblasts and osteoclasts secrete chemokines that recruit these cancer cells, subsequently triggering bone destruction while promoting cancer cell proliferation and differentiation (Hiraga et al., 2012). Among these processes, the RANK/RANKL/OPG axis and canonical Wnt signaling, among others, regulate osteoclast and osteoblast activity (Kenkre and Sbassett, 2018). Furthermore, tumor cell-derived factors such as parathyroid hormone-related protein (PTHrP) and interleukin-11 (IL-11) promote osteoclast activity by altering the RANKL/OPG ratio. During increased osteolysis, tumor growth-promoting factors such as bone morphogenetic proteins (BMPs), insulin-like growth factor-1 (IGF-1), and transforming growth factor-beta1 (TGF-β1) are released from the bone matrix (Clezardi et al., 2007; Clines and Aguise, 2008; Haider et al., 2022). Notably, bone-colonizing breast cancer cells further upregulate osteogenic transcription factors like Runx2, promote the release of bone growth factors and cytokines, accelerate tumor growth, and form a vicious cycle (Song and Wei Cli, 2022). Osteoclasts are key drivers of osteolytic breast cancer metastasis; thus, in addition to conventional radiotherapy and chemotherapy, standard care includes drugs that inhibit excessive bone resorption, such as bisphosphonates and the anti-RANKL antibody denosumab (D'Oronzo et al., 2019). However, these approaches merely mitigate symptoms without addressing metastatic causes.

MiRNAs, as small non-coding RNAs, play a crucial role in regulating the complex cellular crosstalk within the bone microenvironment—a system shaped by secreted factors, signaling pathways, and epigenetic mechanisms—and in establishing and maintaining the bone metastatic environment during the metastatic cascade (Zoni Evan der Pluijm and van der Pluijm, 2016). Researchers have found that miR-20a-5p, derived from exosomes of MDA-MB-231 cells, enhances osteoclast activity and promotes their proliferation and differentiation by targeting the expression of *SRCIN1*—a negative regulator of malignant phenotypes and breast cancer progression (Guo et al., 2019; Grasso et al., 2019). Additionally, miR-141 and miR-219 have been shown to reduce osteoclast activity and diminish osteoblastic breast cancer bone metastasis *in vivo* (Ell et al., 2013). Further studies have revealed that in breast cancer cells, miR-218-5p directly targets two key inhibitors of the Wnt signaling pathway—Sclerostin (*SOST*) and secreted frizzled-related protein 2 (*sFRP2*)—thereby activating Wnt signaling, promoting osteoclast differentiation, and accelerating breast cancer bone metastasis (Taipaleenmäki et al., 2016). It is noteworthy that miR-218 also disrupts bone matrix homeostasis through dual mechanisms: on one hand, cancer cell-secreted miR-218 directly acts on osteoblasts to suppress type I collagen expression; on the other hand, intracellular miR-218 upregulates inhibin βA, which in turn interferes with the processing and deposition of type I collagen (Liu et al., 2018). These direct and indirect effects collectively exacerbate the osteolytic vicious cycle, creating a favorable environment for breast cancer cell colonization in bone tissue. These findings demonstrate that miRNAs can regulate the process of breast cancer bone metastasis through multi-target and multi-pathway mechanisms, highlighting their potential value as therapeutic targets.

## 4 Roles of miRNAs in breast cancer bone metastasis

Numerous studies have shown that miRNAs regulate the intricate communication between bone cells and breast cancer tumor cells, with different functional regulations by miRNAs modulating the vicious cycle of bone metastasis (Table 1) (Croset et al., 2018; Cai et al., 2018). Clinically, alterations in miRNA expression profiles strongly correlate with disease progression and clinical outcomes in breast cancer patients, making them candidate biomarkers for risk stratification, prognosis, and disease monitoring (Swellam et al., 2021).

**TABLE 1:** Roles of miRNAs in Breast Cancer Bone Metastasis.

miRNA	Role	Target genes or pathways	References
miR-21	OncomiR	*SMAD7* and *MSH2*	Chen et al. (2013)
		*TPM1*	Zhu et al. (2007)
miR-10b	OncomiR	*TWIST1*	Ma and Teruya-Feldstein Jweinberg (2007), Croset et al. (2014)
miR-224	OncomiR	*RKIP*	Huang et al. (2012)
		*CASP9*	Zhang et al. (2019)
miR-214-3p	OncomiR	*TRAF3*	Liu et al. (2017)
miR-214	OncomiR	p53	Wang et al. (2015)
miR-665	OncomiR	*NR4A3*	Zhao et al. (2019)
miR-218-5p	OncomiR	Wnt signaling pathway	Taipaleenmäki et al. (2016)
miR-155	OncomiR	*SOCS1* and *C/EBPβ*	Jiang et al. (2012)
miR-30a	TsmiR	*ZEB2*	di Gennaro et al. (2018)
miR-429	TsmiR	*CrkL*	Zhang et al. (2020a)
miR-203	TsmiR	SNAI2	Ding et al. (2013)
miR-31	TsmiR	*SATB2*	Luo et al. (2016)
miR-146	TsmiR	EGFR	Hurst et al. (2009)
	TsmiR	NF-κB	Bhaumik et al. (2008)
miR-1976	TsmiR	*PIK3CG*	Wang et al. (2020a)

### 4.1 OncomiRs in breast cancer bone metastasis

#### 4.1.1 miR-21

In breast cancer, miR-21 is overexpressed and promotes the metastasis of breast cancer cells to the bone, leading to poor prognosis for patients (Sah et al., 2015). Mechanistically, miR-21 drives tumor progression by downregulating *SMAD7* and *MSH2* in the TGF-β pathway while upregulating human epidermal growth factor receptor 2, thereby functioning as an oncogene (Chen et al., 2013; Gong et al., 2011). The tumor suppressor gene *TPM1*, a direct target of miR-21, inhibits anchorage-independent growth when overexpressed in MCF-7 breast cancer cells (Zhu et al., 2007). Other studies have found that lysophosphatidic acid receptor 1 (LPA1), a metastasis-driving GPCR, upregulates oncogenic miR-21 via PI3K/ZEB1 to promote breast cancer invasion and bone colonization; miR-21 inhibition blocks LPA1-mediated migration and reduces metastatic burden *in vivo* (Sah et al., 2015). Therefore, targeting multiple points in the miR-21 pathway may prevent metastatic cells from migrating to the bone.

#### 4.1.2 miR-10b

Highly expressed in metastatic breast cancer cells, miR-10b actively regulates cancer cell migration and invasion, establishing its role as a critical metastasis-associated oncomiR (Ma and Teruya-Feldstein Jweinberg, 2007). Research demonstrates that *TWIST1* promotes breast cancer bone metastasis by binding the miR-10b promoter to induce its expression, which suppresses HOXD10 and activates the metastasis-promoting gene *RHOC*, thereby increasing tumor burden and bone destruction (Ma and Teruya-Feldstein Jweinberg, 2007; Croset et al., 2014). Clinically, serum miR-10b levels are significantly elevated in breast cancer patients with confirmed bone metastasis (sensitivity: 76.9%, specificity: 97.9%) compared to non-metastatic cases, highlighting its potential as a novel biomarker for diagnosing and detecting breast cancer bone metastasis (Dwedar et al., 2021; Roth et al., 2010).

#### 4.1.3 miR-224

Overexpression of miR-224 enhances the metastatic capacity of breast cancer cells. In MDA-MB-231 breast cancer cells, miR-224 overexpression silences the tumor suppressor RKIP, thereby activating pro-metastatic genes (*CXCR4*, *MMP1*, *OPN*) that mediate bone-specific metastasis through enhanced invasion and osteoclast activation (Huang et al., 2012). Furthermore, miR-224 directly suppresses caspase-9 (*CASP9*) by targeting its mRNA, and knockdown of miR-224 in MDA-MB-231 cells significantly restores CASP9 protein levels, thereby inhibiting cell proliferation, migration, and invasion, suggesting preclinical drug candidate for breast cancer patients (Zhang et al., 2019).

#### 4.1.4 miR-214-3p

miR-214-3p, derived from osteoclasts, is an important regulator in breast cancer bone metastasis. Metastatic cancer cells can induce excessive osteoclast formation and overactive bone resorption, leading to osteolytic bone metastasis (OBM). Elevated miR-214-3p expression in osteoclasts, which was observed in bone tissues of breast cancer patients with OBM and corroborated in osteoclasts from breast cancer xenograft (BCX) models, directly targets *TRAF3* to suppress its expression, thereby enhancing osteoclast differentiation and bone-resorbing activity that drives OBM progression (Liu et al., 2017). Moreover, miR-214 directly targets p53 to promote breast cancer cell invasion, and overexpression of p53 reverses this oncogenic effect, suggesting miR-214 as a potential therapeutic target for inhibiting invasion in breast cancer (Wang et al., 2015).

#### 4.1.5 Other OncomiRs

miR-665 is significantly upregulated in breast cancer tissues and promotes cancer cell proliferation, invasion, and metastasis by inhibiting *NR4A3* to activate the MEK signaling pathway, thereby functioning as an oncomiR in breast cancer progression (Zhao et al., 2019). Serum miR-218-5p levels are markedly elevated in patients with bone metastasis compared to non-metastatic cases, suggesting its pro-metastatic role in skeletal dissemination. The pathological elevation of miR-218-5p activates the Wnt signaling pathway, thereby enhancing the metastatic properties of breast cancer cells and exacerbating cancer-induced osteolytic disease (Taipaleenmäki et al., 2016). miR-155 is identified as the most commonly associated with breast cancer (Grimaldi et al., 2020). miR-155 upregulates HK2 through dual mechanisms: suppressing *SOCS1* to activate STAT3-mediated transcription and inhibiting *C/EBPβ* to block miR-143-dependent translational repression, thereby driving oncogenic phenotypes in tumor cells (Jiang et al., 2012). Similarly, within the bone microenvironment, miR-16 promotes the osteoclast activity and osteolytic bone destruction induced by breast cancer metastasis (Kitayama et al., 2021). Interestingly, during the early stages of breast cancer bone metastasis, miR-662 inhibits the differentiation of bone-resorbing osteoclasts, thereby concealing the presence of metastatic foci; overexpression of miR-662 ultimately leads to the development of overt osteolytic metastases (Puppo et al., 2023). Moreover, whether metastasis-promoting miRNAs known to facilitate dissemination to other organs, such as miR-141 [which promotes breast cancer brain metastasis (Debeb et al., 2016)], also participate in the initiation and progression of bone metastasis remains to be clarified.

### 4.2 TsmiRs in breast cancer bone metastasis

#### 4.2.1 miR-30 family

The miR-30 family, comprising miR-30a, miR-30b, miR-30c, miR-30d, and miR-30e, acts as an effective suppressor of breast cancer bone metastasis (Croset et al., 2018). High expression of miR-30a significantly improves survival in triple-negative breast cancer patients. Researchers have further demonstrated that p53 suppresses breast cancer cell invasion and dissemination by activating miR-30a expression, which subsequently inhibits the EMT-related transcription factor *ZEB2* (di Gennaro et al., 2018). In xenograft mouse models of breast cancer stem cells, miR-30 overexpression effectively reduces bone metastasis (Ouzounova et al., 2013). Overall, the regulatory role of the miR-30 family in breast cancer bone metastasis involves multiple critical pathways, providing a theoretical basis for using miR-30 family members to inhibit breast cancer bone metastasis (Croset et al., 2018).

#### 4.2.2 miR-429

As a member of the miR-200 family, miR-429 acts as a tumor suppressor that inhibits cancer progression (Zhang et al., 2016). Compared with primary breast cancer tissue, miR-429 is downregulated in the bone tissue of patients with metastatic breast cancer, suggesting a negative correlation between miR-429 levels and bone metastasis (Zhang X. et al., 2020). However, miR-429 has also been observed at high levels in bone tissue samples from breast cancer patients. Subsequent overexpression of miR-429 in MDA-MB-231 cells significantly suppressed tumor cell invasion (Ye et al., 2015; Zhang L. et al., 2020). Furthermore, miR-429 directly suppresses v-Crk sarcoma virus CT10 oncogene homolog-like (*CrkL*) expression, thereby inhibiting osteoclast-mediated bone resorption and reducing local bone destruction and distant bone metastasis (Zhang X. et al., 2020).

#### 4.2.3 miR-203

miR-203 is notably downregulated in highly metastatic breast cancer cells. Research has shown reveals that TGF-β signaling induces transcriptional repression of miR-203 through direct binding of SNAI2 to the miR-203 promoter, thereby promoting EMT and metastatic progression in breast cancer (Ding et al., 2013). Compared with healthy bone, miR-203 levels are lower in bone tissue from patients with breast cancer bone metastases. In MDA-MB-231 cells, elevated miR-203 expression downregulates cell motility-related genes (*ROCK*, *CD44*, and *PTK2*), reducing primary tumor size and spontaneous metastasis, including bone lesions (Taipaleenmäki et al., 2015). Thus, modulating miR-203 expression may serve as a potential therapeutic approach for metastatic breast cancer patients.

#### 4.2.4 miR-31

miR-31 functions as a multifaceted tumor suppressor, coordinating inhibition of cell cycle progression. Upregulating miR-31 significantly decreases migration and invasion in MDA-MB-231 and MCF-7 cells (Luo et al., 2016). Compared with healthy breast tissue, miR-31 expression is reduced in breast cancer tissue (Gharib et al., 2022), with low expression correlating with adverse pathological features such as bone metastasis (Vimalraj et al., 2013). Notably, the miR-31-p specifically suppresses Dicer expression in MCF-7 cells (Chan et al., 2013). Separately, in triple-negative breast cancer miR-31 acts as a metastasis suppressor by directly targeting *SATB2*, thereby inhibiting tumor cell migration and invasion (Luo et al., 2016).

#### 4.2.5 miR-146

miR-146a/miR-146b exhibits therapeutic potential for suppressing breast cancer metastasis. In MDA-MB-231 cells, miR-146 significantly downregulates epidermal growth factor receptor (EGFR) expression, inhibiting breast cancer cell invasion and migration *in vitro* and mitigating bone destruction (Hurst et al., 2009). Additionally, miR-146a/miR-146b negatively regulates NF-κB activity in highly metastatic breast cancer cell lines, impairing migratory capacity and confirming its tumor-suppressive role (Bhaumik et al., 2008). Consequently, miR-146 has been proposed as a diagnostic and prognostic biomarker for breast cancer bone metastasis.

#### 4.2.6 Other TsmiRs

Research has revealed that miR-1976 expression is lower in bone metastatic breast cancer cells than in primary tumors. Moreover, suppression of miR-1976 promotes epithelial-mesenchymal transition and cancer stem cell properties by targeting *PIK3CG* (Wang J. et al., 2020). Another study demonstrated that within the bone microenvironment, miR-133a and miR-223 inhibit osteoclast activity and bone destruction induced by breast cancer metastasis (Kitayama et al., 2021). During the early stages of breast cancer bone metastasis, miR-24-2-5p suppresses tumor cell dissemination in bone tissue and impedes the differentiation of precursor cells into mature osteoclasts, exerting a protective effect (Puppo et al., 2024).

## 5 miRNA-mediated regulation of key genes in the bone microenvironment during breast cancer bone metastasis

### 5.1 Runx2

Runx2, abnormally expressed in bone-metastatic tumors, serves as a critical regulator of osteogenesis and metastasis in human malignancies including breast cancer (Vishal et al., 2017). Evidence suggests that Runx2 is highly expressed in metastatic breast cancer cells and plays a significant role in regulating breast cancer progression (Chang et al., 2014). Multiple miRNAs have been shown to modulate Runx2 in breast cancer bone metastasis. miR-135 and miR-203 reduce breast cancer bone metastasis by directly targeting and downregulating *Runx2* expression (Taipaleenmäki et al., 2015). miR-3960 targets and downregulates *Hoxa2*, a repressor of Runx2 expression, thereby alleviating its inhibitory effect on Runx2 and promoting BMP-2-induced osteoblast differentiation (Hu R. et al., 2011). Moreover, miR-204 and its homolog miR-211 directly target *Runx2*, suppressing its expression and thereby inhibiting osteogenesis (Huang et al., 2010). miR-153 directly inhibits *Runx2*, thereby suppressing breast cancer cell proliferation, migration, and invasion, establishing the miR-153/Runx2 regulatory axis as a potential therapeutic target (Zuo et al., 2019). These findings collectively confirm Runx2’s pivotal role in breast cancer-mediated bone metastasis.

### 5.2 c-Myc

c-Myc participates in regulating diverse biological functions, including the growth, proliferation, and differentiation of aggressive breast cancer cells, acting as a potent activator of oncogenic transcription (Obaya et al., 2002; Gao et al., 2023). Studies reveal that c-Myc is highly expressed in triple-negative breast cancer cell lines. Let-7a downregulates c-Myc expression by binding to its 3′UTR, thereby inhibiting the proliferation, migration, and invasion of MDA-MB-231 cells (Du et al., 2019). Knockdown of c-Myc significantly reduces the expression of miR-4723-5p, which in turn decreases breast cancer initiation and metastasis (Jin et al., 2022). The *MYC* proto-oncogene, overexpressed in triple-negative breast tissues, cooperates with DNA methyltransferase 3A (*DNMT3A*) to induce promoter methylation, downregulating miR-200b expression and promoting EMT (Pang et al., 2018). Additionally, miR-3189-3p exerts anti-tumor activity by targeting transforming regulatory proteins, impairing c-Myc translation, and inhibiting skeletal metastasis, providing novel therapeutic insights (Vittori et al., 2022).

### 5.3 Cyclin D1

Cyclin D1 becomes overexpressed following genomic rearrangement and functions as a crucial oncogene in breast cancer pathogenesis (Mohammedi et al., 2019; Arnold and Papanikolaou, 2005). miR-373, a well-characterized oncomiR, influences patient prognosis by modulating cyclin D1 (Bakr et al., 2021). Similarly, miR-425-5p overexpression upregulates Cyclin D1 protein levels and activates PI3K/AKT signaling, both of which contribute to breast cancer development (Zhang LF. et al., 2020). In breast cancer cells, the miR-17/20 cluster demonstrates an inverse relationship with cyclin D1 protein levels, suppressing its translation via 3′UTR binding and inhibiting cancer cell proliferation (Yu et al., 2008). Moreover, miR-1250-5p expression is significantly reduced in triple-negative breast cancer cells. Restoring miR-1250-5p decreases cyclin D1 protein levels, induces apoptosis, and inhibits epithelial-mesenchymal transition, thereby exerting tumor-suppressive effects (Shuaib and Kumar, 2023). These findings suggest that strategic upregulation or downregulation of specific miRNAs represents an effective approach to modulate Runx2, c-Myc, and cyclin D1 expression, potentially enabling novel therapeutic strategies against breast cancer bone metastasis.

### 5.4 Other potential biomarkers

Interleukins (ILs) play crucial regulatory roles in multiple stages of the breast cancer bone metastasis cascade (Haider et al., 2021). Among them, IL-1β, IL-6, IL-8, IL-11, and IL-17 have been extensively validated as mediators of bidirectional communication between bone cells and breast cancer cells, making them potential therapeutic targets (Haider et al., 2021; Zhou and Tulotta Cottewell, 2022; Shibabaw and Teferi Bayelign, 2023). miRNAs also influence bone metastasis progression by regulating these IL-mediated signaling pathways. For example, miR-520b suppresses breast cancer cell migration through targeting the HBXIP/IL-8 regulatory network (Hu N. et al., 2011). Notably, studies have identified miR-204, miR-211, and miR-379 as inhibitors of TGF-β-induced IL-11 production in bone metastatic breast cancer cells; these miRNAs directly target *IL-11* expression by binding to its 3′UTR (Pollari et al., 2012). Further research revealed that miR-124, which is significantly downregulated in breast cancer bone metastases, inhibits osteoclastogenesis and suppresses bone metastasis through direct targeting of its downstream gene, *IL-11* (Cai et al., 2018). Collectively, these findings suggest that miRNA-mediated regulation of interleukins is a critical molecular mechanism involved in the bone microenvironment during breast cancer bone metastasis.

The transcription factor SOX9, markedly upregulated in breast cancer patient samples, serves as a key regulator of breast cancer cell survival and metastasis (Ma et al., 2020; Chao et al., 2022). Studies have demonstrated that downregulation of miR-134-3p and miR-224-3p increases SOX9 levels, thereby promoting breast cancer progression (Chao et al., 2022). Similarly, research has shown that miR-215-5p targets the oncogenic transcription factor *SOX9*, effectively inhibiting breast cancer cell invasion, metastasis, and disease progression (Gao et al., 2019). Additionally, miR-133 modulates breast cancer tumorigenesis and metastasis via targeting *SOX9* (Wang et al., 2018).

Furthermore, miRNAs interact with long non-coding RNAs (lncRNAs) through various mechanisms, including acting as competing endogenous RNA sponges, regulating miRNA degradation, mediating intrachromosomal interactions, and modulating epigenetic elements, ultimately influencing tumor initiation and progression (Bhattacharjee et al., 2023). In breast cancer, inhibition of lncRNA *PANDAR* reduces cancer cell proliferation and invasion (Li and Su Xpan, 2019), while lncRNA *SPRY4-IT1* promotes breast cancer cell proliferation and stemness by targeting miR-6882-3p (Song et al., 2019). Further research demonstrates that lncRNA *SNHG3* modulates osteogenic differentiation of bone marrow mesenchymal stem cells during bone metastasis via the miR-1273g-3p/BMP3 axis (Sun et al., 2022), and *TRG-AS1* competitively binds miR-877-5p to upregulate *WISP2*, thereby inhibiting bone metastasis (Zhu et al., 2023).

## 6 miRNAs as potential biomarkers for the detection and treatment of breast cancer bone metastasis

Bone involvement in metastatic breast cancer presents a significant clinical challenge, with approximately 65%–70% of advanced-stage patients developing bone metastases (Salvador and Llorente, 2019). Early prevention and accurate diagnosis are therefore critical for improving survival outcomes. Current imaging modalities, including bone scintigraphy, positron emission tomography-computed tomography (PET-CT), and magnetic resonance imaging (MRI), exhibit comparable diagnostic performance for bone metastasis detection, yet their specificity and sensitivity remain suboptimal due to multifactorial limitations (Kundaktepe et al., 2021). Key limitations impairing their performance include variable detection of micrometastases, challenges in differentiating malignant from benign bone conditions, and the high cost or radiation exposure of certain techniques—prompting the exploration of auxiliary diagnostic techniques. miRNAs demonstrate relative stability and tissue specificity in physiological fluids, with detectable alterations in serum, plasma, breast ductal fluid, and circulating exosomes of breast cancer patients (Ying et al., 2023; Do Canto et al., 2016). Changes in miRNA expression correlate with disease status and clinical outcomes, suggesting their potential as non-invasive biomarkers for “liquid biopsy,” an approach that provides advantages including low invasiveness, cost-effectiveness, and technical simplicity (Haider et al., 2022; Cai et al., 2015; Ochiya and Hashimoto Kshimomura, 2025).

**FIGURE 3:**
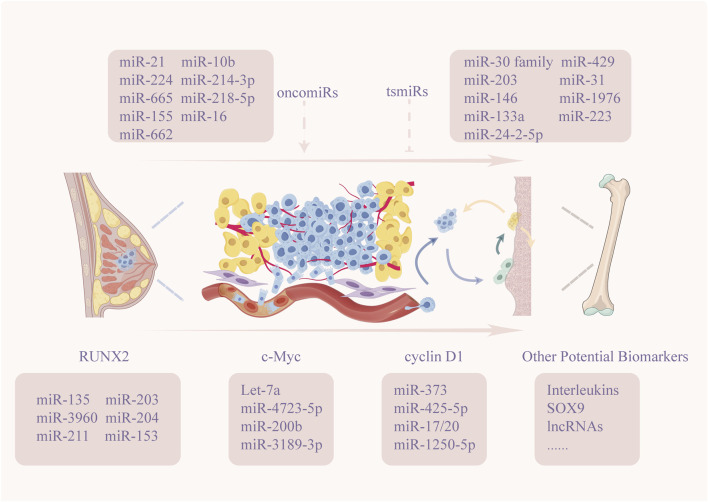
miRNA Biomarkers in Breast Cancer Bone Metastasis.

By comparing serum miRNA profiles of healthy individuals and breast cancer patients, researchers have identified a combination of miR-1246, miR-1307-3p, miR-4634, miR-6861-5p, and miR-6875-5p that detects breast cancer with high sensitivity, specificity, and accuracy (Shimomura et al., 2016). Moreover, numerous clinical studies (Nassar and Nasr Rtalhouk, 2017; Chiorino et al., 2023; Jordan-Alejandre et al., 2023) support the potential of miRNAs in early breast cancer detection. Our investigations further indicate that, in the complex interactions between breast cancer cells and the bone microenvironment, miRNAs may serve as potential biomarkers for detecting bone metastases. We have summarized the key miRNAs that, under experimental intervention, directly or indirectly affect the progression of bone metastasis (Figure 3). In clinical studies, serum levels of miR-129, miR-24-2-5p, miR-662, and miR-10b show promise as biomarkers for early diagnosis of bone metastasis in breast cancer patients (Puppo et al., 2023; Puppo et al., 2024; Wang C. et al., 2020; Zhao et al., 2012). However, studies have indicated that miR-21 acts as a non-specific molecule broadly involved in systemic inflammatory responses and multi-organ metastatic processes (Jenike and Ehalushka, 2021); therefore, its solitary detection in body fluids lacks organ specificity. Combining miR-21 detection with bone-specific miRNAs or serum markers indicative of osteogenesis or osteolysis, and validating these combinations in large-scale prospective cohorts, may establish such non-specific miRNA markers as adjunctive tools for assessing the risk of breast cancer bone metastasis. Furthermore, findings based on laboratory studies and small-scale clinical validation may exhibit significant variability. Therefore, large-scale, fixed-population microRNA screening with extended follow-up durations needs to be conducted. Clinically valuable diagnostic screening should be performed according to breast cancer subtype specificity, combined with other imaging modalities and biological markers (Ochiya and Hashimoto Kshimomura, 2025), to detect metastatic lesions at earlier stages and implement more effective therapeutic interventions.

Inhibition of oncomiRs or restoration of tsmiRs has emerged as a potential therapeutic strategy in cancer treatment. Two primary miRNA-based approaches exist: (1) miRNA replacement therapy, which utilizes miRNA mimics to restore or overexpress tsmiR function; and (2) miRNA inhibition, employing inhibitors to suppress or downregulate oncomiRs (Abou Madawi et al., 2024; Mollaei and Safaralizadeh Rrostami, 2019). Recent advances have overcome miRNA delivery barriers through engineered carriers with optimized size, shape, structure, chemistry, and functionality, enabling efficient targeted delivery for cancer gene regulation to modulate gene expression and treat cancer (Tian et al., 2024). Researchers have developed polyethyleneimine-modified magnetic nanoparticle carriers to deliver miR-21, miR-145, and miR-9, achieving targeted *in situ* tumor delivery and effectively inhibiting tumor growth (Yu et al., 2016). In another study, researchers designed ultra-small iron oxide nanoparticles conjugated with anti-miR-10b antagonists (MN-anti-miR10b), successfully resulting in complete and sustained regression of local lymph node and distant metastatic lesions without detectable systemic toxicity (Le Fur et al., 2021). Further studies demonstrated that MN-anti-miR10b treatment reduced the stem-cell-like characteristics of breast cancer cells, making it a promising therapeutic option for stem-like breast cancer subtypes (Halim et al., 2024). Similarly, branched polyethyleneimine (PEI 25k), a commonly used gene vector, has been modified with alendronate groups as bone-targeting moieties to construct a targeted delivery system for miR-34a to breast cancer bone metastases, achieving significant antitumor effects in metastatic lesions (Han et al., 2023). Notably, a bone-targeting polymeric nucleic acid delivery carrier (ALN-Pabol) chieved direct skeletal delivery of miR-339, offering a promising strategy against bone-metastatic breast cancer (Li et al., 2025).

## 7 Conclusion

Over the past decade, RNA-based medicine has received increasing attention, with non-coding RNAs including miRNAs emerging as promising candidates for therapeutic development alongside mRNA-based approaches. Although no miRNA-targeted drugs have yet received clinical approval, several miRNA-based compounds are undergoing preclinical evaluation and clinical trials, showing encouraging progress in cancer treatment applications. Recent advances in transcriptomic research have further reinforced the diagnostic and prognostic value of miRNAs, particularly as predictors of therapeutic response and biomarkers for breast cancer bone metastasis formation and progression. The growing complexity of breast cancer research has spurred extensive investigations into miRNA-mediated regulatory networks, revealing differential expression patterns and mechanistic insights into miRNAs associated with tumor growth and skeletal dissemination. In metastatic breast cancer cells, changes such as miRNA silencing, translational blockade, miRNA modification, or overexpression suggest new possibilities for delivering miRNAs or inhibiting miRNA synthesis to modulate key metastatic oncogenes—a promising direction for future breast cancer bone metastasis therapies.

Capitalizing on the stability of miRNAs in biofluids and their pivotal roles in bone metastasis, current research explores the possibility of modulating breast cancer-derived miRNA expression to alter tumor cell phenotypes or disrupt bone microenvironment interactions. Such approaches may pave the way for developing precision therapies and preventive strategies. However, inherent biological challenges persist, including off-target effects, tissue specificity limitations, and delivery system inefficiencies inherent to short non-coding RNA molecules. Overcoming these barriers is critical for advancing miRNA-based therapeutics, particularly for unmet clinical needs like metastatic bone disease. Future efforts should resolve these challenges to translate miRNA research into clinically viable treatments for breast cancer bone metastasis.
